# The quest for reproducibility

**DOI:** 10.1590/0100-3984.2021.54.2e2

**Published:** 2021

**Authors:** Felipe Kitamura, Flávia Paiva Proença Lobo Lopes

**Affiliations:** 1 Head of Artificial Intelligence at Diagnósticos da América (Dasa), Neuroradiologist at Escola Paulista de Medicina da Universidade Federal de São Paulo (EPM-Unifesp), São Paulo, SP, Brazil. Email: kitamura.felipe@gmail.com.; 2 Clinical Research Coordinator at Dasa, Visiting Professor in the Graduate Program of Radiology at the Universidade Federal do Rio de Janeiro (UFRJ), Rio de Janeiro, RJ, Brazil.

Reproducibility is critical to advancing the sciences, including the medical sciences^(1)^. It also has a tremendous impact on clinical practice. For example, imagine reading a magnetic resonance imaging (MRI) scan of a patient with a brain tumor. It is essential to determine whether the apparent diffusion coefficient (ADC) and perfusion maps have changed from the previous scan. To make that comparison, we must guarantee that the methods (ADC and perfusion mapping in MRI) are reproducible. Otherwise, the studies are not comparable. From the literature and our own experience, we know that the same MRI scanner may not produce the same result after some time has passed. It is also known that different MRI scanners may produce different results. Therefore, we cannot know whether the ADC or perfusion has changed over time. This topic has long been debated among researchers, and, in the last decade, it has gained a new facet: machine learning (ML) research. As expected, ML research has its own issues, one of which is reproducibility.

According to Beam et al.^(1)^, a study is deemed reproducible if “given access to underlying data and analysis code, an independent group can obtain the same result observed in the original study.” A similar concept is replicability. A study is replicable if “an independent group studying the same phenomenon reaches the same conclusion after performing the same set of experiments or analyses after collecting new data.” On the basis of those definitions, we observe that data and analysis code are the main factors posed to influence ML research reproducibility.

The article by Siqueira et al.^(2)^, published in the previous issue of **Radiologia Brasileira**, compares the influence that factors related to data acquisition (physician and equipment manufacturer) and factors related to analysis code (preprocessing techniques, like normalization) have on the pixel intensities of ultrasound images. The findings of those authors are of great importance because they show how brittle the standardization process can be, depending on acquisition factors. If these data were used to train ML models, we would not be surprised if we got low performance metrics (garbage in, garbage out). In this context, the Siqueira et al.^(2)^ article brings to light a vital issue related to using ultrasound images to create ML models.

The Radiological Society of North America has embraced the importance of reproducibility by creating the Quantitative Imaging Biomarkers Alliance^(3)^, the objective of which is to reduce variability “across devices, sites, patients, and time.” In addition, the Sociedade Paulista de Radiologia (Paulista Society of Radiology) has also created the Grupo de Imagem Quantitativa - GIQ (Quantitative Imaging Group) to study this topic^(4)^, and that may have a major impact on patient diagnosis in clinical practice.

Notwithstanding the influence of data acquisition on reproducibility, we also emphasize the importance of open communication of complete source code to foster reproducibility in ML research^(5)^.

## References

[1] Beam AL, Manrai AK, Ghassemi M (2020). Challenges to the reproducibility of machine learning models in health care. JAMA.

[2] Siqueira GLG, Sousa RP, Olinda RA (2021). Proposal for computer-aided diagnosis based on ultrasound images of the kidney: is it possible to compare shades of gray among such images?. Radiol Bras.

[3] RSNA Quantitative imaging biomarkers alliance.

[4] SPR, Grupo de estudos de imagem quantitativa da SPR (Giq) https://www.spr.org.br/evento/37/grupo-de-estudos-de-imagem-quantitativa-da-spr-giq/sobre-o-giq.

[5] Kitamura FC, Pan I, Kline TL (2020). Reproducible artificial intelligence research requires open communication of complete source code. Radiology: Artificial Intelligence.

